# A pilot study to examine the association between COX-2 rs5275 polymorphism and the response to repetitive transcranial stimulation in schizophrenia

**DOI:** 10.1038/s41537-023-00386-5

**Published:** 2023-09-08

**Authors:** Pingping Wang, Xiaoni Guan, Xiuru Su, Fengchun Wu, Meihong Xiu

**Affiliations:** 1 Neurology Department, Xuan Wu Hospital of Capital Medical University, Beijing, China; 2 Peking University HuiLongGuan Clinical Medical School, Beijing HuiLongGuan Hospital, Beijing, China; 3 Hebei Rongjun Hospital, Baoding, China; 4 Department of Psychiatry, The Affiliated Brain Hospital of Guangzhou Medical University, Guangzhou, China; 5 Department of Biomedical Engineering, Guangzhou Medical University, Guangzhou, China; 6 Guangdong Engineering Technology Research Center for Translational Medicine of Mental Disorders, Guangzhou, China

**Keywords:** Schizophrenia, Biomarkers

## Abstract

High frequency (HF)-rTMS has been shown to improve cognitive functions in patients with schizophrenia (SCZ). This study aimed to investigate whether COX-2 rs5275 variants were associated with cognitive improvements following rTMS treatment in patients with SCZ. Forty-eight hospitalized patients with SCZ were assigned to the neuronavigation HF-rTMS group and 28 patients to the sham group over left DLPFC for 1 month. Cognitive function was evaluated using the repeatable battery for the assessment of neuropsychological status (RBANS) at weeks 0 and 4. COX-2 rs5275 polymorphism was genotyped by a technician. At baseline, C allele carriers showed better cognitive performance relative to patients with TT homozygote. Additionally, C allele carriers had greater improvement in memory from the follow-up to baseline following rTMS stimulation, while patients with the TT genotype showed no significant improvement in memory index. More importantly, we found that COX-2 rs5275 was correlated with the response to rTMS after controlling for the covariates. This study data indicate that COX-2 rs5275 was associated with improvements in immediate memory after HF-rTMS treatment in patients with SCZ. rTMS shows an effect on memory only in C allele carriers, but not in those with the TT genotype.

## Introduction

Patients with schizophrenia (SCZ) are usually associated with multiple domains of cognitive deficits, which are the core symptoms of SCZ^1^. There is clear evidence that cognitive impairments are present at different stages of SCZ, from prodrome to multi-episode stage, and more severe in chronic patients^2^. Longitudinal studies (at least 6-month follow-up) have reported significant associations between cognition and community outcomes in SCZ^3^. Antipsychotic drugs are the main treatment for SCZ and are effective in alleviating positive symptoms, however, they have a minimal effect on cognitive deficits and their treatment remains unsatisfactory^4,5^. Therefore, there is a need to further explore novel therapeutic approaches to enhance cognitive functions in patients with SCZ.

The important pathogenic role of chronic inflammation in the disease course of SCZ has been documented, where cytokines are considered to be essential factors related to the onset and progression of cognitive dysfunction in patients^6^. SCZ has been shown to be correlated with dysfunctions or deficits in all components of the immune system: from innate to adaptive immunity, and from humoral to cellular immunity^7–10^. Evidence supports that microglial activation and kynurenine pathway-related brain abnormalities are the underlying pathological mechanisms for cognitive decline in SCZ^11^. Some pro-inflammatory components from immune or glial cells induce the form of plasticity that regulates pre- and postsynaptic functions^12^. There are three cyclooxygenases (COX) isoenzymes that catalyze the metabolism of arachidonic acid to PGs^13^. COX-2 is constitutively expressed in the central nervous system (CNS). It not only interacts with neurotransmitters such as serotonin, acetylcholine, and glutamate but also participates in the regulation of the immune system and CSN inflammation through the action of prostaglandins^14^. The COX-2 -PGE2 signal pathway is well known to suppress natural killers, T cells, and type-1 immunity, but promote type-2 immunity^15^. Thus, as part of the immune system, the COX-2 gene may contribute to the balance of different immune cell types in certain diseases^16^ and thus serve as a predictive biomarker for the detection and treatment of clinical symptoms.

Recently, repetitive transcranial magnetic stimulation (rTMS) has been reported to be efficacious for alleviating auditory hallucinations and negative symptoms in patients with SCZ^17–20^. In particular, a previous meta-analysis supported that high frequency (HF) rTMS targeting the left DLPFC enhanced cognitive functions in SCZ, especially working memory^21^. Meanwhile, rTMS has been reported to induce long-lasting effects on memory in patients with dementing disorders and healthy individuals^22–24^. The potential mechanism of rTMS-enhancing cognitive performance in SCZ may be related to the neuronal priming, driving of oscillatory activity, and synaptic neuroplastic changes in directly stimulated DLPFC or other relevant areas by the magnetic fields^25^. Specifically, noninvasive brain stimulation can produce “neuroenhancement” when applied to the brain^26^. However, some clinical trials have revealed that the administration of HF-rTMS has no effect on cognitive performance in patients with SCZ^27–29^, suggesting that the effects of HF-rTMS on cognition in SCZ were inconsistent and mixed.

Current understanding of the underlying mechanism of rTMS treatment may be attributed in part to the modulatory of local cortical plasticity and/or remote neural circuitry^30^. Several biological pathway biomarkers associated with synaptic plasticity, particularly metaplasticity have been reported to influence the response to rTMS in neuropsychiatric disorders^31,32^. Based on the relationship between COX-2 and cognitive function, we hypothesized that COX-2 rs5275 polymorphism was associated with cognitive improvements after HF-rTMS over left DLPFC for 4 consecutive weeks in SCZ patients. To test this hypothesis, this study was designed to investigate whether cognitive improvements were associated with COX-2 rs5275 polymorphism after controlling for covariates.

## Methods

### Patients

The study protocol was approved by the Institutional Review Board of Hebei Rongjun Hospital. Each patient provided written, informed consent to participate in this clinical trial.

One hundred and thirty-one SCZ inpatients were recruited in the hospital. Recruited patients should meet the following recruitment criteria: (1) SCZ diagnosed by DSM-IV using the SCID-I/P; (2) between 30 and 70 years; (3) male inpatients; (4) without MECT therapy in the past 24 months; (5) at least 5 years of illness; and (6) taking a stable dose of antipsychotic drugs for more than 6 months. The exclusion criteria included the following: (1) any other psychiatric disorder diagnosed using SCID-I/P; (2) pregnant; (3) substance abuse or drug dependence; (4) history of epilepsy or family history of epilepsy; (5) with risk of suicide or self-harm; (6) switching the type of antipsychotic or changing the dose during rTMS treatment; and (7) comorbid central nervous system disorder by verbally asking the patients whether they had central nervous system disorders, such as brain pathology, severe headache or severe head injury.

### Treatment protocol

The samples were from two randomized, blinded, controlled trials. The duration of rTMS treatment was 4 weeks for a total of 20 sessions. A computer program was used to generate a random number list. Patients with SCZ were assigned to either the active 10 Hz rTMS group or the sham group according to the randomized sequence. Researchers, patients, and raters remained blinded to the trial grouping throughout the trial.

Neuronavigation rTMS was administrated with the MagStim Rapid Stimulator as the protocols described in our previous studies^33,34^. Each patient was treated on the left DLPFC once a day, five times a week, for a total of four consecutive weeks [23]. Patients in the sham group received the same study procedures as 10 Hz stimulation, except that the sham coil (P/N:3910-00) looked identical to the active coil and patients could not distinguish whether they were assigned to the 10 Hz group or the sham group. The stimulations over left DLPFC occurred at a power of 110% of MT^35^.

### Outcomes

The outcome measure was cognitive functions assessed using the repeatable battery for the assessment of neuropsychological status (RBANS) at weeks 0 and 4 after treatment. It was assessed by the nurses who were blinded to the randomization number. RBANS consists of the total score and five index scores: immediate memory, visuospatial/constructional, attention, language, and delayed memory index scores^36^.

### Genotyping

Blood was drawn from patients to separate white blood cells and used to extract germline DNA using standard procedures. Rs5257 polymorphism in the *COX-2* gene was genotyped using the MALDI-TOF MS platform (Sequenom, CA, USA), following the standard procedure.

### Statistical analysis

Intent-to-treat (ITT) analyses were conducted in this study. Missing outcome data after 3 weeks of treatment were filled in using the last observed non-missing data. Differences in clinical characteristics and cognitive functions between real and sham rTMS groups or between genotype groups were performed by ANOVA analysis. The primary hypothesis was tested in the real rTMS group. The impact of rs5257 polymorphism on cognitive functions was examined by the repeated-measures (RM) analysis of covariance (ANCOVA). In the RM-ANOVA model, the within-subject factors included the Time factor (two levels, baseline and week 4) and the genotypic factor (two levels). The primary hypothesis was tested in the interactive effect between Time and Treatment. We were more focused on the interaction effect of Time and genotypic group. If it was significant, the difference between the two genotypes at week 4 was compared by ANCOVA with the baseline scores as covariates. In addition, the improvements in cognitive functions were also compared between the two genotypic groups in patients by using the Wilcoxon signed-rank test. Exploratory regression analysis was adopted to examine the predictive factors for the improvement of neurocognitive functioning.Two-tailed *p*-value was used and the significance level was set at 0.05.

### Human and animal rights

No animals were used in this research. All human research procedures followed were in accordance with the standards set forth in the Declaration of Helsinki principles of 1975, as revised in 2008 (http://www.wma.net/en/20activities/10ethics/10helsinki/).

## Results

### Baseline demographic and neurocognitive functioning

RM-ANCOVA analysis revealed a significant interaction effect of time and stimulation group on immediate memory (*p* < 0.05). Of the initial 131 recruited patients, the patients without COX-2 rs5275 genotype data were removed, and a total of 76 patients were included in the following analysis. Forty-eight patients were in the HF-rTMS group and 28 patients were in the sham group. Genotypic frequencies for COX-2 rs5275 were CC (*n* = 8), CT (*n* = 24), and TT (*n* = 44). Because of the insufficient number of patients with the CC genotype for the following analysis, the CT heterozygote was combined with CC homozygotes to C allele carriers.

Table 1 shows the RBANS total score and its five subscores of SCZ patients among rs5275 genotypes. There were no significant differences in age, education years, hospital times, the dose of antipsychotics, duration of illness, and onset age between C allele carriers and patients with TT homozygotes (all *p* > 0.05). However, we found a significant difference in the RBANS total score between genotypes (*p* = 0.04). Patients with the CC genotype showed higher cognitive performances compared with T carriers (61.0 ± 12.6 vs. 55.0 ± 11.0).

**Table 1:** Demographic and clinical data among patients with TT genotype and C allele carriers at baseline.

	TT genotype (*n* = 44)	C allele carriers (*n* = 32)	*X*^2^ or *F*(*p*)
Age (yr)	56.0 ± 8.2	56.3 ± 6.5	0.04(0.85)
Education (yr)	7.5 ± 2.0	8.0 ± 2.0	1.1(0.29)
Age of onset (yr)	20.9 ± 1.7	20.9 ± 1.8	0.0001(0.99)
Duration of illness(yr)	35.0 ± 7.4	35.8 ± 6.3	0.2(0.63)
Hospital time	6.2 ± 2.9	6.0 ± 2.3	0.1(0.74)
Dose of antipsychotics	449.8 ± 227.9	421.0 ± 229.2	0.3(0.62)
*RBANS scores*			
Immediate memory	51.1 ± 12.0	54.1 ± 13.1	1.0(0.32)
Visuospatial/constructional	68.7 ± 12.3	70.0 ± 11.1	0.2(0.69)
Language	75.5 ± 15.2	80.6 ± 14.9	1.9(0.17)
Attention	66.2 ± 13.6	64.4 ± 9.8	0.4(0.53)
Delayed memory	58.8 ± 17.9	65.4 ± 18.5	2.3(0.14)
Total score	55.0 ± 11.0	61.0 ± 12.6	4.5(0.04)

*yrs* years, *RBANS* repeatable battery for the assessment of neuropsychological status.

### Comparison of stimulation efficacy between different genotypes in the rTMS treatment group

First, the RM-ANCOVA analysis including all patients (*n* = 76) revealed a significant interaction effect of the stimulation group (real vs. sham groups) on the immediate memory index of RBANS (*F* = 4.0, *p* = 0.049).

**Fig. 1:**
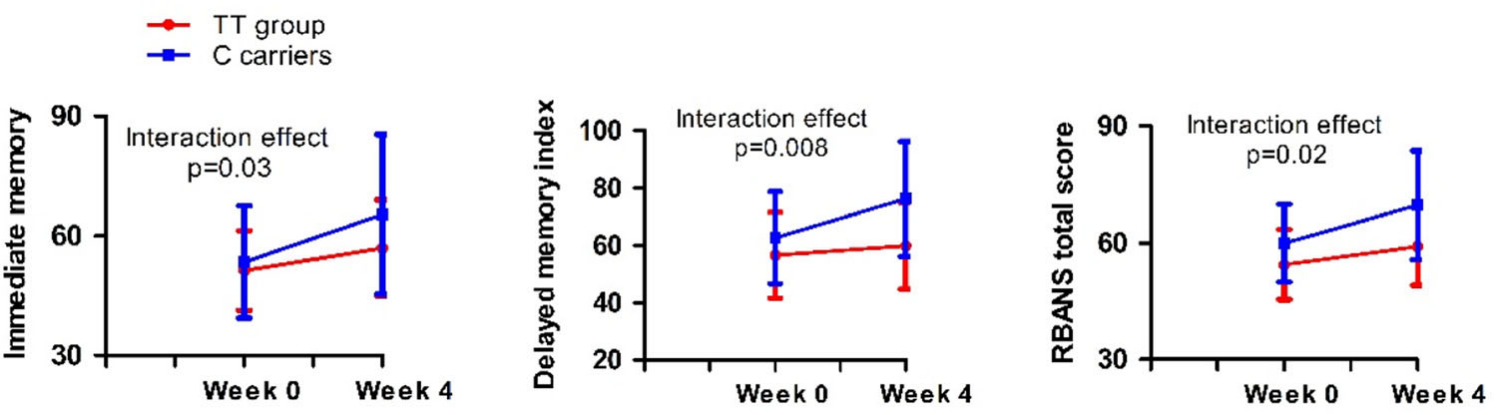
Interaction effects of group and time on cognitive funtion. RM-ANCOVA analysis showed significant genotypic group × time interaction effects on immediate memory index, delayed memory index, and RBANS total score (all *p* < 0.05).

RM-ANCOVA analysis was performed to investigate the improvements in cognitive functions between the genotypes in the real rTMS group. We found significant genotypic group × time interaction effects on immediate memory index, delayed memory index, and RBANS total score in the rTMS treatment group (all *p* < 0.05) (Table 2) (Fig. 1). In addition, as shown in Table 2, the main effects of group on delayed memory index and RBANS total score and main effects of time on immediate memory, attention, delayed memory, and RBANS total score were significant (all *p* < 0.05). There was no significant interaction effect of genotypic group × time on RBANS scores in the sham group (*F* = 0.22, *p* = 0.64).

**Table 2:** Cognitive functioning at baseline and after treatment with HF rTMS among the rs5275 genotypes.

	Baseline (*n* = 44)	Week 4 (*n* = 44)	Group effect *F*(*p*)	Time effect *F*(*p*)	Group × Time *F*(*p*)
*Immediate memory index*			1.6(0.21)	39.1(<0.001)*	5.0(0.03)*
TT	51.3 ± 10.1	56.9 ± 12.3			
C carriers	53.4 ± 14.7	65.3 ± 20.3			
*Visuospatial*/*constructional index*			0.04(0.84)	4.8(0.04)*	0.002(0.96)
TT	68.9 ± 11.1	73.9 ± 13.5			
C carriers	69.8 ± 5.6	74.6 ± 21.4			
*Language index*			1.1(0.30)	4.1(0.05)	0.8(0.37)
TT	73.9 ± 16.3	77.4 ± 16.4			
C carriers	79.9 ± 13.2	81.3 ± 14.3			
*Attention index*			0.01(0.92)	0.3(0.60)	1.4(0.24)
TT	66.5 ± 14.3	64.7 ± 15.6			
C carriers	64.8 ± 10.0	65.5 ± 9.8			
*Delayed memory index*			5.0(0.03)*	20.9(<0.001)*	7.8(0.008)*
TT	56.6 ± 15.0	59.9 ± 15.9			
C carriers	62.7 ± 16.7	76.3 ± 21.9			
*RBANS total score*			5.9(0.02)*	48.1(<0.001)*	6.0(0.018)*
TT	54.4 ± 9.3	59.1 ± 10.3			
C carriers	59.9 ± 11.2	69.7 ± 14.8			

*p < 0.05.

### Improvements in cognitive functions among genotypes in the HF-rTMS group

Further analyses between the two genotypes in the active group suggested that there were significant differences in the increases in immediate memory, delayed memory, and total RBANS score between the two genotypes (*Z* = −2.3, *Z* = −2.1, *Z* = −2.0, all *p* < 0.05). The increases in immediate memory delayed memory, and total RBANS score in C allele carriers were observed to be significantly greater than those with TT homozygotes (all *p* < 0.05). When the cognitive improvements were used as the dependent variable and *rs5275* was used as the independent variables and age, duration of illness, educational levels, and cognitive performance at baseline as covariates, regression analysis revealed that genotype was a significant predictive factor for immediate memory improvement (beta = 0.28, *t* = 2.28, *p* = 0.026) (*R*^2^ = 0.17).

## Discussion

We found that rs5275 polymorphism in the COX-2 gene was correlated with cognitive improvements following HF-rTMS treatment for 4 weeks in SCZ, after adjusting for the confounding factors. In addition, Rs5275 C allele carriers showed greater improvements in cognitive functions.

We found that SCZ patients with the TT genotype showed worse cognitive performance compared to C allele carriers. Rs5275 variant located in the 3’UTR of the COX-2 gene has an impact on COX-2 transcriptional activities and expression levels^37^. A growing body of studies suggests that activated proinflammatory cytokines modulate synaptic efficacy and the intrinsic excitability of neurons in the brain and influence cognitive performance, memory, and behavior by immune-triggered neuroplasticity^12^. COX-2 enzyme, inducibly expressed in microglia and astrocytes in response to proinflammatory molecules^38,39^, is also involved in the regulation of immune response and cognitive function in patients with SCZ. The frontal cortex and hippocampus, regions of the brain related to cognition and memory in the brain, express COX-2 in postsynaptic dendritic spines and excitatory terminals of cortical and spinal cord neurons and are regulated by synaptic activity^40,41^. Animal models of long-term potentiation (LTP) and long-term depression (LTD) revealed a modulatory role of COX-2 in LTP function^42–44^, and pharmacological COX-2 inhibition directly attenuated LTP in the CA1 region of the hippocampus^45^, suggesting a fundamental role of COX-2 enzyme in learning and memory^46^.

To our best knowledge, this study reports for the first time an association between COX-2 rs5275 polymorphism and cognitive improvements after 4 weeks of rTMS treatment in SCZ. Since COX-2 is involved in regulating inflammation and immune responses, our findings provide further evidence that inflammation and certain modulations of the immune system are the underlying mechanisms of cognitive decline in SCZ. Interestingly, the COX-2 enzyme was reported to be up-regulated by HF stimulation, similar to the induction of LTP. More importantly, clinical trials have also found that COX-2 inhibitors modulate immune function and show effects on cognition in patients with SCZ^14,47,48^. In addition to the above modulations, COX-2 is also induced by the trans-synaptic activation of the dopaminergic, serotoninergic, and cholinergic neurotransmitter systems, and its decreased activity may contribute to the development of cognitive decline^49,50^. Rs5275 C allele carriers showed greater improvement in cognitive functions after HF-rTMS treatment, whereas patients with the TT genotype showed no significant improvements. The underlying mechanism of better improvements in C allele carriers following HF-rTMS may be due to its modulation of NMDAR-dependent LTP and LTD synaptic plasticity. Upon stimulation, neuronal COX-2 enzymes were activated in response to synaptic excitation to produce the predominant COX-2 metabolite in the neurons, which in turn stimulates the release of neurotransmitters, such as glutamate. The different responses among the genotypes may be due to the rTMS-induced persistent changes in COX-2 production-related signals and the intensity of the LTP/LTD-like effects after stimulation between TT homozygotes and C allele carriers.

It should be noted that in this study, time may have an effect on the cognitive functions of patients with SCZ. As shown in Table 2 for the cognitive scores at baseline and follow-up assessments for both the rs5275 TT and rs5275 C carriers for the active HF-rTMS, the cognitive functions were increased in both the rs5275 TT and rs5275 C carriers. However, the effects of time (i.e., repeated cognitive assessments) were much lower than the effects of group (i.e., rs5275 polymorphism) and the interaction time*genotypic group was significant. Thus, the effect of group (i.e., genotype) was stronger than time in patients receiving HF-rTMS. Given that the C carriers had a better performance at baseline as well compared to the TT homozygotes and the increases in cognitive functions in C carriers were greater than those with TT homozygotes, we speculate that C carriers may function better at baseline, be less affected by this disorder, and therefore achieve greater cognitive improvement after rTMS treatment. Nonetheless, this is only our speculation, and our study does not clearly show whether these differences were driven by rTMS or by group differences in cognitive ability.

Several limitations were noted in the present study. First, it is a pilot study. The small sample size is addressed in the present study, which reduces the statistical power of this study. Second, this study only recruited long-term hospitalized male inpatients on stable antipsychotic medication. Thus, the current findings have limited generalizability in clinical applications and our results cannot be generalized to female patients. Third, only one single nucleotide polymorphism within the COX-2 gene was analyzed. Several other functional polymorphisms have been identified within the COX-2 gene, including rs20417 in the promoter region, rs689466, and rs3218625 in the coding region^37^. It remains unclear whether other polymorphisms can interact with rs5275 to impact the response to rTMS in SCZ. In addition, the interrelationship between the COX-2 gene and other immune-related genes was not measured in the present study. Forth, COX-2 levels in CSF or blood were not measured and analyzed in this study. Therefore, we did not know the impact of the rs5275 C/T variant on its expression or levels. Further studies should analyze COX-2 levels that would lend consistency to all the reported findings in our study.

In conclusion, the rs5275 variant in the COX-2 gene may be involved in the response to neuronavigation HF-rTMS stimulation in the long-term hospitalization of patients with SCZ. The present study provides further evidence for immune-related molecules in the clinical response to rTMS stimulation in SCZ. However, given the limitations stated above and the possible involvement of complex neuroplasticity-related biological pathways not yet studied in this study, additional replications using larger sample sizes are warranted to better understand the potential role of COX-2 in the short and long-term rTMS treatment outcomes.
