# Author Correction: A pilot study to examine the association between COX-2 rs5275 polymorphism and the response to repetitive transcranial stimulation in schizophrenia

**DOI:** 10.1038/s41537-024-00431-x

**Published:** 2024-01-15

**Authors:** Pingping Wang, Xiaoni Guan, Xiuru Su, Fengchun Wu, Meihong Xiu

**Affiliations:** 1 Neurology Department, Xuan Wu Hospital of Capital Medical University, Beijing, China; 2 Peking University HuiLongGuan Clinical Medical School, Beijing HuiLongGuan Hospital, Beijing, China; 3 Hebei Rongjun Hospital, Baoding, China; 4 Department of Psychiatry, The Affiliated Brain Hospital of Guangzhou Medical University, Guangzhou, China; 5 Department of Biomedical Engineering, Guangzhou Medical University, Guangzhou, China; 6 Guangdong Engineering Technology Research Center for Translational Medicine of Mental Disorders, Guangzhou, China

**Keywords:** Schizophrenia, Biomarkers

Correction to: *Schizophrenia* 10.1038/s41537-023-00386-5; published online 08 September 2023

In this article, the affiliation details for Author Meihong Xiu were incorrectly given as ‘Hebei Rongjun Hospital, Baoding, China’ but should have been ‘Peking University HuiLongGuan Clinical Medical School, Beijing HuiLongGuan Hospital, Beijing, China’. The original article has been corrected.

